# Utilisation of Machine Learning Approaches Improves RNA-Seq Transcriptome Analyses in Alzheimer’s Disease Brain

**DOI:** 10.1007/s12031-025-02469-7

**Published:** 2026-01-15

**Authors:** Yuning Cheng, Kristina Santucci, Yulan Gao, Konii Takenaka, Grace Lindner, Si-Mei Xu, Michael Janitz

**Affiliations:** School of Biotechnology and Biomolecular Sciences, University of New South Wales, Sydney, NSW 2052 Australia

**Keywords:** Alzheimer’s disease, Transcriptome, Differential expression, Gene ontology, Machine learning

## Abstract

Alzheimer’s disease (AD) is a neurodegenerative disorder that progressively deteriorates a person’s memory, as well as their ability to think and move. It has been reported to be the most common cause of dementia. Alterations in gene expression have been increasingly recognised as key contributors to the onset and progression of AD, driving interest in transcriptomic approaches to better understand the disease at a molecular level. The development of machine learning (ML) approaches in transcriptomics have been rapid in the past decade, and this advancement can be applied to the study of AD transcriptomes. An ML program that enhances the alignment data through filtering out low confidence splice junction reads, Splam, has been developed by Chao et al. (2023). However, this program has not been utilised and assessed in the transcriptomic study of a complex neurological disease such as AD. This study investigates both the transcriptome of AD brain and the potential of an ML program to enhance alignment-stage data quality and influence downstream analyses. Using the Integrative Genomics Viewer, a selection of filtered reads was visualised, uncovering the types of splice junction reads Splam discards to refine the alignment data. From the differential expression (DE) analysis, we found a higher number of DE transcripts using ML-filtered data compared to unfiltered data, potentially unmasking aspects of AD brain DE profile obscured by alignment noise. The gene loci expressing those transcripts were also determined to be more AD-relevant by comparing these findings with external studies, and contribute to more related gene ontology enrichment terms. We identified gene loci expressing transcripts of interest shared between ML-filtered and unfiltered data, as this consistency in detection suggests that these genes are robust candidates for downstream analyses and biomarkers in AD.

**Supplementary Information:**

The online version contains supplementary material available at 10.1007/s12031-025-02469-7.

## Introduction

Alzheimer’s disease (AD) is a progressive neurodegenerative disorder that primarily affects memory, thinking, and motor behaviour (DeTure and Dickson 2019). It has been reported to be the most common cause of dementia, typically occurring in older adults over the age of 65 (Ferreira et al. 2014). AD is characterised by the accumulation of amyloid plaques and tau tangles in the brain, leading to the death of nerve cells and brain tissue shrinkage (DeTure and Dickson 2019; Breijyeh and Karaman 2020). Neurodegeneration can also occur due to large scale atrophy in the form of neuronal and synaptic loss, as well as other factors such as neuroinflammation and oxidative stress (Breijyeh and Karaman 2020). Although there is no cure, treatments including anti-amyloid drugs and immunotherapy are available to help manage symptoms and slow disease progression (Yiannopoulou and Papageorgiou 2020).

The progression of AD is typically classified into four main stages: pre-clinical, mild stage of AD, moderate stage of AD, and severe stage of AD or dementia (Breijyeh and Karaman 2020; Monteiro et al. 2023). Along with these classifications of disease progression, the Clinical Dementia Rating (CDR) scale is commonly used to assess and assign the severity of dementia in individuals with AD (Morris 1997). The CDR evaluates six domains of functioning: memory, orientation, judgment and problem solving, community affairs, home and hobbies, and personal care (Morris 1997). Each domain is rated on a scale from 0 to 3, and a global score is derived to reflect overall dementia severity (Morris 1997). Extension to CDR stages four and five have been proposed for “profound” and “terminal” stages of dementia (Dooneief et al. 1996).

The disease progression of AD has been found to be reflected in distinct gene expression changes across different tissues and disease stages, as revealed by transcriptomic analyses (Huseby et al. 2022; Wu et al. 2023). Transcripts expressed by *CCDC92*, *GRIA4*, *HDAC7*, and *IFITM3* were reported to be significantly DE in AD blood transcriptome, as well as documented to be involved in other neurological disorders or pathways contributing to AD pathology, such as immunity (Gupta et al. 2020; Hur et al. 2020; Abdullah et al. 2022). To further demonstrate that changes in the transcriptomic profile can serve as indicators of AD onset and progression, Huseby et al. (2022) utilised a panel of transcripts from genes including *MRPL51*, *NDUFA1*, and *NDUFS5* to distinguish AD samples from healthy controls, achieving an AUC of around 80%.

The transcriptome is one of the most studied biological datasets in the field of complex disease due to its dynamic nature sensitive to disease progression (Ziemann et al., 2019). However, several aspects of transcriptomic analysis—such as the large volume of data, high dimensionality, and biological noise—present significant challenges to conventional analytical approaches. Over the past decade, the application of machine learning (ML) in biomedical research has expanded rapidly, driven by the increasing availability of high-dimensional omics datasets, particularly transcriptomic and gene expression data (Cheng et al. 2024). This is evident in the exponential growth in the number of papers published on deep learning in bioinformatics (Min et al. 2016). ML offers distinct advantages in this context, particularly in its ability to efficiently process large-scale data, uncover complex non-linear patterns, and perform robust data integration, such as from one dataset to another with higher accuracy and scalability (Cheng et al. 2024). These capabilities are especially impactful in the study of complex, heterogeneous diseases including AD. ML models have been used to identify AD or other disease-specific gene expression signatures, identify potential diagnostic biomarkers, predict disease progression, and stratify patients into subtypes based on genetic information (Park et al. 2020; Warnat-Herresthal et al. 2020; Ahmed et al. 2022; Cheng et al. 2024).

Although the repertoire of ML programs developed for transcriptome analysis is rapidly expanding, there has not been a comprehensive evaluation of their performance on datasets beyond those used by the original developers. This gap makes it difficult to fully assess the potential of these tools across diverse biological contexts. We address this gap by using Splam (Chao et al. 2023), an ML-based tool that enhances transcriptomic alignment data by filtering out low confidence splice junction reads, to investigate whether ML can improve downstream analyses, specifically in the context of AD. Alternative splicing is a regulatory mechanism that allows a single gene to produce multiple mRNA isoforms, increasing transcriptomic and proteomic diversity (Wang et al. 2015). This process involves the selective inclusion or exclusion of specific exons during pre-mRNA processing, enabling the generation of distinct transcripts from the same gene based on the cellular context (Wang et al. 2015). In addition, we employed rMATS-turbo (Wang et al. 2024), a tool for detecting differentially expressed (DE) alternative splicing events, to explore the relationship between splicing and transcript expression in AD.

To evaluate the practical impact of ML-enhanced alignment data, we compared the results of downstream analyses based on Splam-modified alignments to those based on unmodified alignments assembled by a conventional alignment tool, STAR (Dobin et al. 2013). This comparison enabled us to assess how—and to what extent—improvements from ML at the alignment stage influence downstream outcomes. By directly contrasting these two alignment approaches, our study aimed to determine whether ML can enhance the sensitivity, specificity, and/or biological relevance of transcriptomic analyses in complex disease contexts.

## Materials and Methods

### Data Download

The RNA-seq dataset utilised in this study was sourced from the Gene Expression Omnibus (GEO) (Clough and Barrett 2016) under accession number GSE53697 in FASTQ format. It comprised 17 samples: eight controls exhibiting no neurofibrillary tangles or plaque pathology and nine advanced AD samples with a CDR between four and five. For comprehensive details on the data acquisition procedures prior to this step, refer to the “Materials and methods” section of the original paper (Scheckel et al. 2016).

### Data Pre-Processing

Quality check using FastQC v0.11.9 (Andrews 2010) was performed on the downloaded dataset to ensure all samples were of sufficient quality. Using Trimmomatic v0.39 (Bolger et al. 2014), adaptor sequences and poor-quality reads in the dataset were removed, following the paired-end sequences protocol on the usadellab website manual (http://www.usadellab.org/cms/?page=trimmomatic). A second quality check with FastQC was conducted on the trimmed data to confirm the quality of the data was maintained.

### Read Alignment

Sequencing reads were aligned to the reference genome using STAR v2.7.9a (Dobin et al. 2013). The run settings were set as recommended by the authors of rMATS-turbo (Wang et al. 2024), a program used downstream to analyse DE alternative splicing events. The genome index required by STAR was generated using the UCSC hg38 reference genome, along with the GENCODE v39 Gene Transfer Format (GTF) annotation file.

### Alignment Filtering of Reads with Low Confidence Splice Junctions

The ML program, Splam v1.0.10 (Chao et al. 2023), was selected for its function of cleaning existing spliced alignments, which potentially improved downstream analyses. Procedures regarding the program were conducted following the recommendations in the program’s documentation (https://github.com/Kuanhao-Chao/splam). The main output for each sample comprised a list of reads containing splice junctions and their confidence scores determined by Splam, as well as a cleaned alignment where reads with low confidence splice junctions were filtered out based on the default threshold of confidence < 0.1. Using the Integrative Genomics Viewer (IGV) (Robinson et al. 2011), a selection of filtered splice junction reads with the lowest confidence scores was visualised and compared.

To evaluate how Splam-filtering influences splicing accuracy, splice sites were pulled from BAM alignments - both pre- and post-filtering - by detecting intron-spanning events in CIGAR codes (Li et al. 2009) using RegTools v1.0.0 (Cotto et al. 2023). Junction coverage was calculated to measure confidence, as weakly supported sites tend to stem from mapping errors. Splice motifs were determined from RegTools annotations to distinguish canonical GT–AG types from non-canonical junctions, which serves as an indicator of biological plausibility (Parada et al. 2014). Intron length distributions were calculated from junction coordinates, flagging those exceeding 100 kb, as such structures are biologically rare and likely to be products of spurious splicing (Mapleson et al. 2018). Junction count per gene was tallied from annotated junctions to estimate splicing noise, since inflated junction counts per gene often reflect misalignment or false positives (Zhang et al. 2018).

### Transcript Assembly and Quantification

On unfiltered and ML-filtered data, transcript assembly and quantification were performed using StringTie v1.3.4d (Shumate et al. 2022). The run settings were set to default. StringTie was used to assemble the transcripts and obtain the structural information of the transcript assembly in the format of GTF files. Using StringTie’s *merge* function, all individual GTF files corresponding to each sample were merged. Transcript abundance was then estimated with StringTie’s *-e* and *-B* options using the merged GTF and alignment files as input. Using a python script “prepDE.py3” provided by the StringTie developers (https://ccb.jhu.edu/software/stringtie/index.shtml?t=manual), read count information was extracted from these estimated transcript abundances.

### Differential Expression Analysis of Transcripts

On unfiltered and ML-filtered data, DE analysis of transcripts was performed using edgeR v4.2.1 (Robinson et al. 2010) and limma v3.60.6 (Ritchie et al. 2015) in R environment v4.2.0 (R_Core_Team 2021). Procedures for DE analysis and related data pre-processing followed the edgeR-limma workflow outlined by Law et al. (2016), with the following modifications. Transcripts that were expressed in only one condition (e.g., present in AD but not in control, or vice versa), referred to as “uniquely expressed” in the respective condition, were excluded. Transcripts with low expression levels—defined as having a total CPM of less than 0.1 across all samples—were discarded. Initially, the significance threshold for DE was set at an adjusted *p*-value < 0.05. Under this criterion, only one DE transcript was identified in the unfiltered data, and none in the ML-filtered. To explore potential DE patterns between control and AD brain despite this limitation, the threshold was relaxed to an unadjusted *p*-value < 0.01.

A comparison between the DE transcripts identified in unfiltered vs. ML-filtered data was conducted. Gene loci expressing DE transcripts shared between the two types of datasets and DE transcripts exclusively found in ML-filtered data based on genomic coordinates were determined.

### Differential Expression Analysis of Alternative Splicing Events

On unfiltered and ML-filtered data, DE analysis of alternative splicing events was performed using rMATS-turbo v4.3.0 (Wang et al. 2024). rMATS-turbo detects splicing events in alignment data and utilises a statistical model for their quantification and the determination of DE. Procedures were conducted on unfiltered and ML-filtered data following the recommendations from the program’s documentation (https://github.com/Xinglab/rmats-turbo). Additionally, gene loci associated with DE splicing events were compared to gene loci associated with DE transcripts to investigate the potential connection between the two types of DE patterns. Shared gene loci between them were then identified.

### Transcripts Uniquely Expressed in Alzheimer’s Disease Brain

In this study, as mentioned in Method section “2.6 Differential expression analysis of transcripts”, transcripts expressed in only one condition (e.g., present in AD but not in control, or vice versa), were termed as “uniquely expressed” in the respective condition. A comparison of the uniquely expressed in AD transcripts identified in unfiltered vs. ML-filtered data was conducted. Gene loci expressing transcripts shared between the two types of datasets and transcripts exclusive to ML-filtered data were determined. A manual literature search was performed on those transcripts exclusive to ML-filtered data with annotated gene loci to determine whether they were biologically relevant to AD pathology.

### Literature Survey of Gene Loci of Interest

A Bash script was developed to perform an automated literature survey on gene loci of interest. The script is designed to retrieve and filter gene-specific information relevant to AD from the NCBI Gene database (https://www.ncbi.nlm.nih.gov/gene/). The workflow comprises the following steps:

Prior to executing the script, the input list of gene symbols was sorted and deduplicated to generate a non-redundant gene set. Each unique gene symbol was then converted to its corresponding NCBI Gene ID, which was required for constructing valid NCBI Gene database URLs, as these URLs utilise Gene IDs rather than gene symbols.

The script used the *curl* command to retrieve the HTML content of each gene-specific NCBI page. This content was parsed using the *grep* command, which searched for manually curated keywords associated with AD pathology. The presence of these keywords indicated potential relevance of the gene to AD. The selected keywords included:

“Alzheimer” — captures Alzheimer’s, Alzheimer-related.

“amyloid” — captures amyloid, amyloid-beta.

“astrocyt” — captures astrocyte, astrocytic.

“cognit” — captures cognitive, cognition.

“dement” — captures dementia, dementia-related.

“memor” — captures memory, memories.

“microgli” — captures microglia, microglial.

“neurodegenerat” — captures neurodegenerative, neurodegeneration.

“neuroinflamm” — captures neuroinflammation, neuroinflammatory.

“neuro” — captures neuron, neuronal.

“plaque” — captures plaque.

“tangl” — captures tangle, tangling.

“tau” — captures tau, tauopathy.

This approach enabled the rapid and automated identification of genes with textual evidence, linking them to AD-related biological processes or pathologies.

Gene loci associated with DE transcripts from unfiltered and ML-filtered data, as well as DE transcripts exclusive to ML-filtered data underwent this procedure.

### Gene Ontology Enrichment Analysis of Gene Loci of Interest

Gene Ontology (GO) enrichment analysis was performed on gene loci associated with DE transcripts detected from unfiltered and ML-filtered data using clusterProfiler v4.4.4 (Wu et al. 2021) in R environment v4.2.0. Using the *enrichGO* function, GO over-representation analysis was conducted to identify enriched biological process terms, applying a significance cut-off of *p*-value < 0.05. The analysis was carried out with reference to org.Hs.eg.db, the Genome-wide annotation for Human (Carlson 2024). Treeplots visualising the GO enrichment results were generated using enrichplot v1.16.1 (Yu 2024) in an R environment.

Gene loci corresponding to DE transcripts exclusively found in ML–filtered data were examined in terms of their contribution to the enrichment of GO terms relevant to AD, through searching with keywords such as “neuro”, “apopto”, and “calcium”, which reflected critical biological processes implicated in AD pathology, followed by manual extraction.

### Functional Assessment of Novel MSTRG Gene Loci

To assess the potential biological relevance of novel DE MSTRG transcripts identified exclusively in the ML-filtered data, functional analyses of coding potential prediction and evolutionary conservation were conducted. For each transcript associated with an MSTRG locus, its corresponding nucleotide sequence was extracted from the reference genome, along with its genomic coordinates (chromosome, start, end, and strand) in the FASTA format.

Coding potential was evaluated using CPC2 (Kang et al. 2017), which predicts the probability that a transcript encodes a protein, using features such as open reading frame (ORF) length, nucleotide composition, and Fickett score. CPC2 was run online at https://cpc2.gao-lab.org with the MSTRG transcript sequences as input. Transcripts were classified as coding or non-coding by CPC2 based on their coding probability scores.

Evolutionary conservation was investigated via BLASTn search of MSTRG transcript sequences in the mouse transcriptome using BLAST + v2.16.0 (Camacho et al. 2009). The mouse transcript reference (GENCODE release M34) was downloaded and converted into a nucleotide BLAST database. BLASTn searches were performed with an E-value threshold of 1 × 10⁻⁵, and for each MSTRG transcript, the best alignments factoring in query coverage length and identity was used as a representative. Transcripts exhibiting significant cross-species similarity were interpreted as showing evidence of evolutionary conservation.

## Results

### Differentially Expressed Transcripts in Control vs. Alzheimer’s Disease Brain

**Fig. 1:**
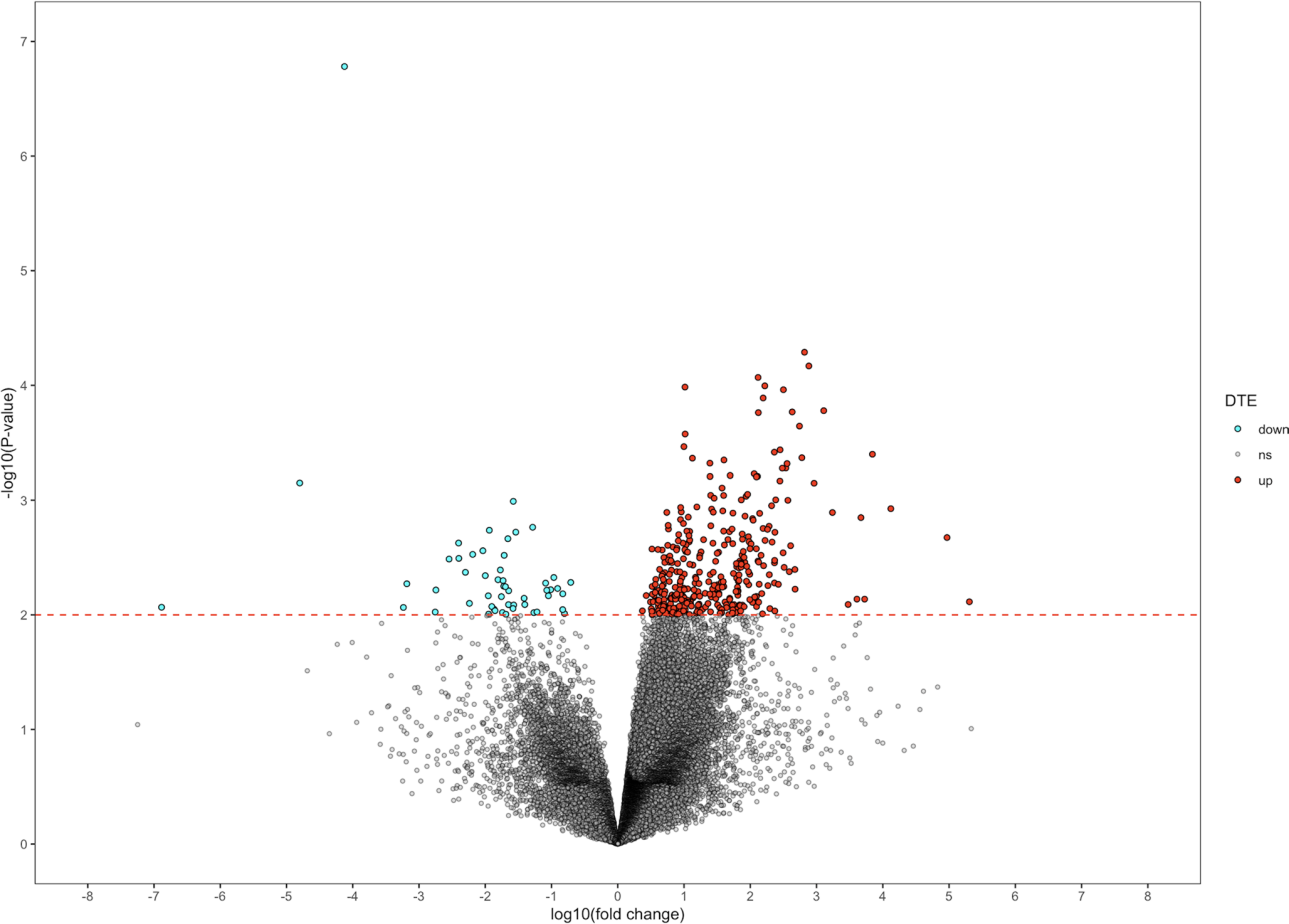
Volcano plot of DE transcripts in control vs. AD brain for unfiltered alignment data. The significance cut-off in the dotted red line is *p*-value < 0.01. Down-regulated transcripts (down) are shown in blue, up-regulated (up) in red, and non-significant (ns) in grey. Figure created using R v4.2.0. DTE – differential transcript expression

DE analysis was performed on transcripts in control vs. AD brain. From unfiltered alignment data, using a stringent cut-off of adjusted *p*-value < 0.05, only one significantly DE transcript down-regulated was identified (Table 1A). The significance cut-off was then set to *p*-value < 0.01 to reduce stringency, yielding 416 significantly DE transcripts, with 53 being down-regulated and 363 being up-regulated (Table 1A). The proportion of transcripts with positive fold-change regarding AD brain was higher in terms of total and DE transcripts in unfiltered data (Fig. 1).

**Table 1:** Number of DE transcripts in control vs. AD brain for (A) unfiltered and (B) machine learning-filtered alignment data

	Adj. *p*-value < 0.05	*p*-value < 0.01
(A)		
Down-regulated	1	53
Non-Significant	60,812	60,397
Up-regulated	0	363
(B)		
Down-regulated	0	35
Non-significant	60,664	60, 163
Up-regulated	0	466

**Fig. 2:**
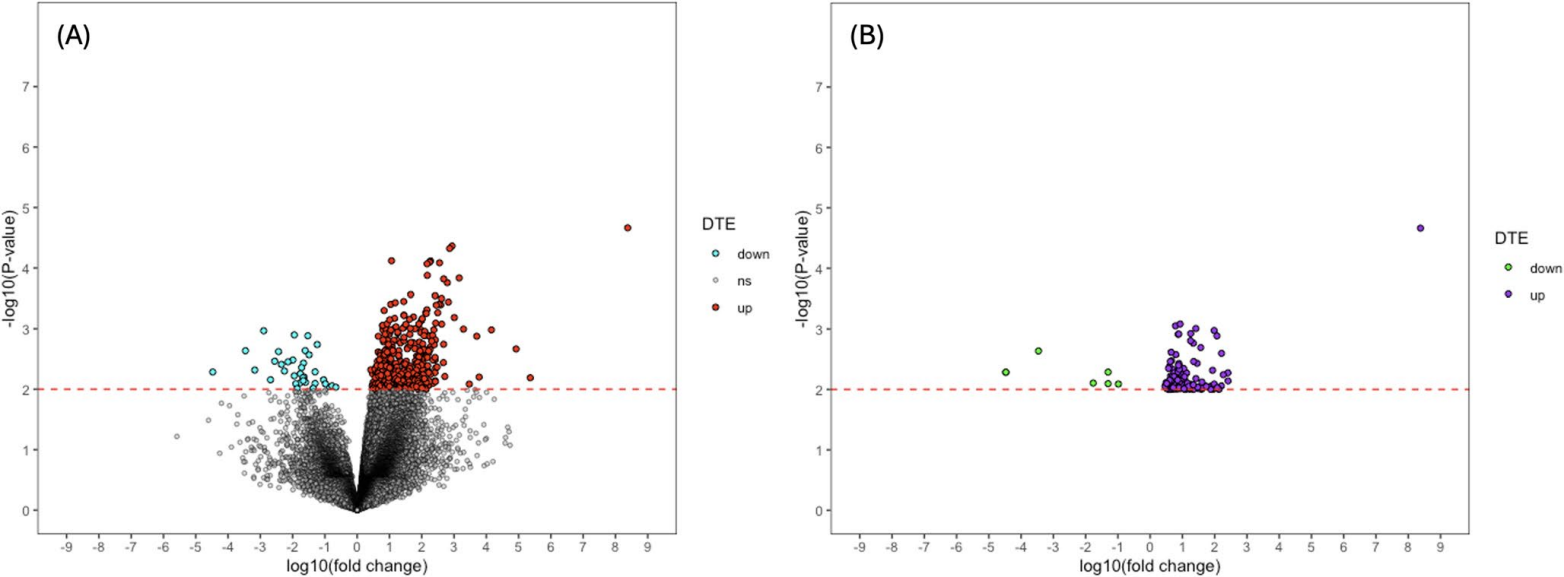
Volcano plot of DE transcripts in control vs. AD brain using machine learning (ML)–filtered alignment data. (A) The entirety of the DE transcripts from ML-filtered data. (B) Subset of (A), representing the 157 DE transcripts exclusively identified through the ML–filtered approach. Figure created using R v4.2.0. DTE – differential transcript expression; up – up-regulated transcripts; down – down-regulated transcript; ns – non-significant transcripts

DE analysis for ML-filtered alignment data by *Splam* exhibited a similar pattern of higher proportion of significantly up-regulated transcripts (Fig. 2A). No significantly DE transcripts were identified with the cut-off of adjusted *p*-value < 0.05 (Table 1B). Notably, when using a less stringent cut-off of non-adjusted *p-*value < 0.01, the total number of DE transcripts was higher in the ML-filtered alignment in comparison to the unfiltered alignment data, being 85 transcripts more (Table 1B). The number of down-regulated transcripts was 35, 18 transcripts less than with unfiltered alignment data, however, there were 103 more up-regulated transcripts (Table 1B), which accounted for the increase in total DE transcripts.

**Fig. 3:**
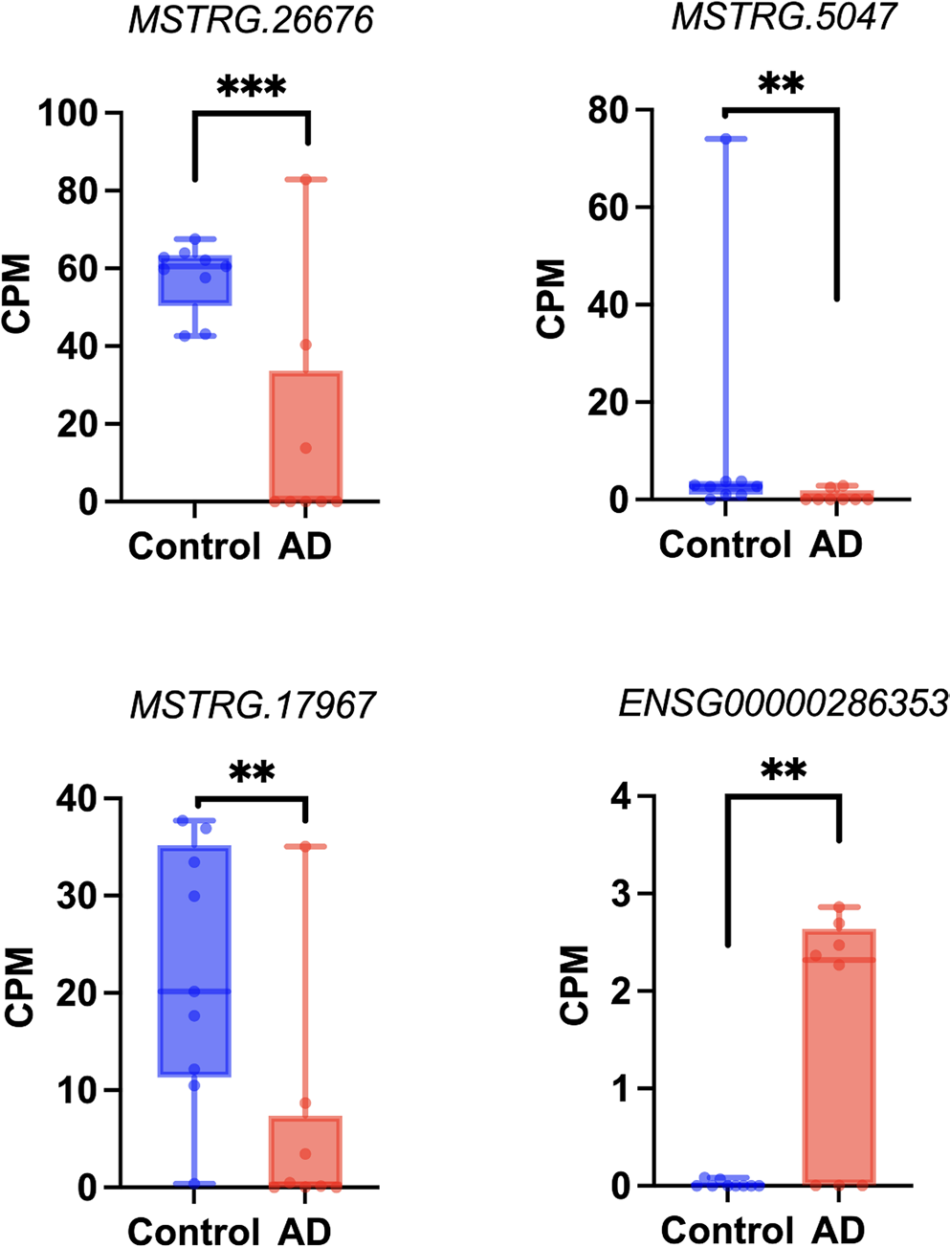
Top four DE transcripts by fold-change in control vs. AD brain exclusive to machine learning-filtered alignment data. Transcripts labelled with a “MSTRG” prefix are novel transcripts determined by the transcript assembler, StringTie. The order of the transcripts from highest to lowest is *MSTRG.26,676* (chr3:54091246–54149904), *MSTRG.5047* (chr10:96518121–96586967), *MSTRG.17,967* (chr18:31846751–31943106), and *ENSG00000286353* (chr3:54057690–54125377). The plot was created using Prism v10.2.0 for Mac, GraphPad Software, San Diego, California USA, www.graphpad.com. ** *p*-value < 0.01, *** *p*-value < 0.001

This difference in the number of DE transcripts was examined, with 157 DE transcripts having been identified to be exclusive to ML-filtered alignment data (Table S1). The distribution of these 157 DE transcripts regarding statistical significance and fold-change is shown in Fig. 2B. Upon further investigation, it was found that 62 out of these 157 transcripts were novel as determined by the transcript assembler, StringTie, by the naming convention of a “MSTRG” prefix (Table S1). Among the top four DE transcripts by fold-change exclusive to ML-filtered approach, three were novel (Fig. 3).

The gene loci of DE transcripts shared between the unfiltered and ML-filtered alignment data were examined. A total of 345 genes were found (Table S2). The consistency in detection suggests that these genes are strong candidates for further downstream analyses.

### Machine Learning Tool, Splam, Removes Potentially Ambiguous Splicing Events Occurring in Intronic or Non-Coding Regions

**Fig. 4:**
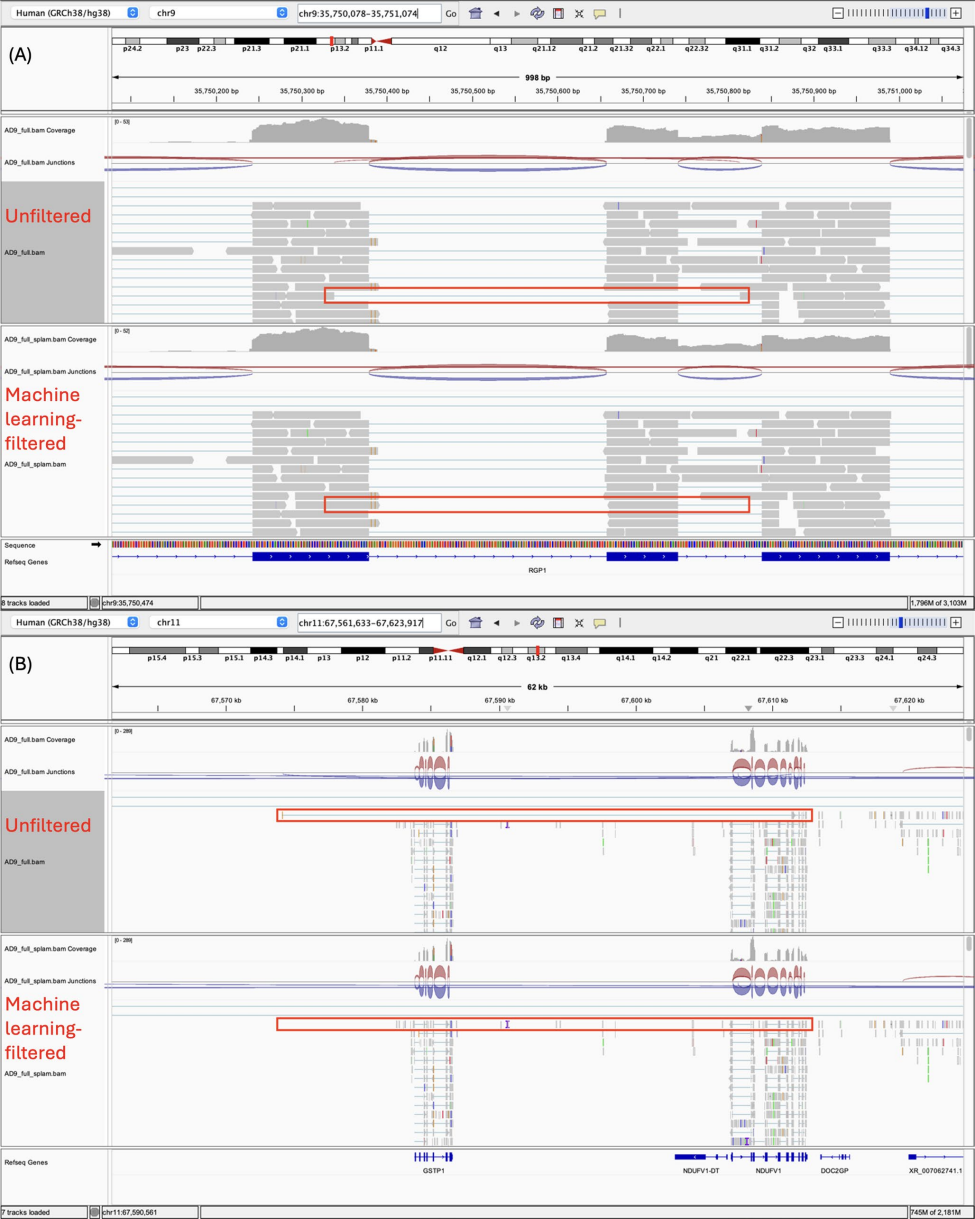
Two examples of splice junction reads (**A**) and (**B**) filtered from the alignment due to low confidence scores from the machine learning (ML) program, Splam, derived from replicate AD9. The IGV images display two tracks for each example: the top track (indicated as unfiltered) shows the unfiltered alignment, while the bottom track (indicated as machine learning-filtered) represents the ML-filtered alignment. Splice junction reads that were filtered out based on low confidence scores are highlighted with red boxes. Both examples illustrate ambiguous splicing events occurring in intronic or non-coding regions

Using Splam, the STAR alignments were modified by filtering out reads containing splice junctions with low confidence scores from the ML program. Using replicate AD9 as an example, out of the 273,871 total splice junction reads, 30,924 splice junction reads were evaluated to have a confidence score of < 0.1, the default filtering threshold of Splam, and were thus discarded from the alignment. The list of splice junction reads and their confidence scores for AD9, determined by Splam, can be found in Table S3. A selection of filtered splice junction reads with the lowest confidence scores were examined visually using IGV. In Fig. 4, two examples demonstrating the filtering of splice junction reads suggesting ambiguous splicing events occurring in intronic or non-coding regions are shown. The filtered splice junction reads may indicate partial retention of both exonic and intronic regions (Fig. 4A), and long spanning events including intronic regions across multiple gene loci (Fig. 4B). Similar observations were made across all biological replicates when examining the splice junction reads that were filtered out of the alignment data based on a low confidence score.

Junction-level quality metrics revealed that for AD9, while the median read support per junction remained stable (1,471 reads before filtering vs. 1,443 reads after filtering), the proportion of well-supported junctions increased substantially, with junctions supported by ≥ 5 reads increasing from 36.98% to 53.38%. The proportion of canonical splice motifs became higher, as canonical GT–AG splice motifs increased from 83.9% without filtering to 97.6% after using Splam, indicating effective removal of non-canonical and likely spurious junctions. Additionally, Splam-filtering reduced the number of intron structures that were perhaps implausible. Median intron length decreased from 2,678 bp to 2,340 bp, and the proportion of very long introns (> 100 kb) decreased from 9.29% to 5.59%. Lastly, junction count per gene was reduced; the mean number of junctions per gene decreased from 15,049 to 11,517, suggesting removal of splicing noise.

### Transcripts Uniquely Expressed in Alzheimer’s Disease Brain

**Fig. 5:**
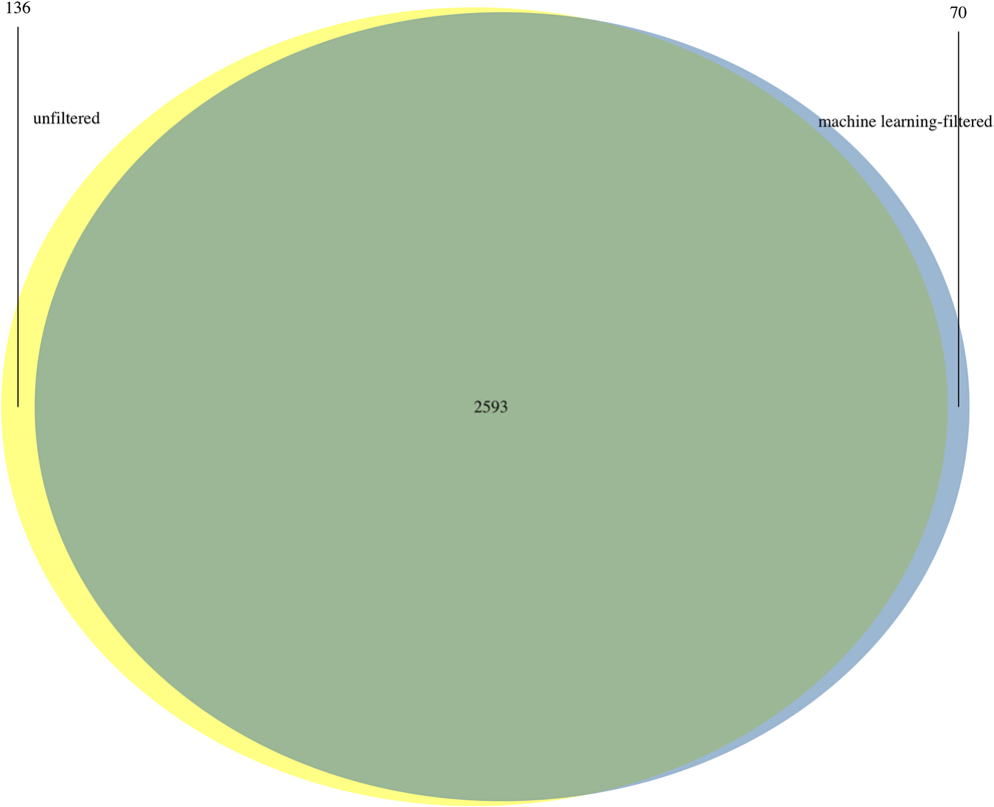
Venn diagram of the number of transcripts uniquely expressed in AD brain for unfiltered and machine learning-filtered alignment data. Figure created using R v4.2.0

In this study, transcripts expressed in AD brain with no expression in controls have been termed “uniquely expressed in AD brain”. After filtering out the splice junction reads with low confidence scores determined by Splam, the number of transcripts uniquely expressed in AD decreased by 66, from 2,729 to 2,663 (Table 2). Examination based on genomic coordinates revealed that 136 of these transcripts were exclusively found in unfiltered alignment data and 70 were exclusive to ML-filtered alignment data (Table 2; Fig. 5). The majority of transcripts exists in both unfiltered and ML-filtered alignment data (Fig. 5).

**Table 2:** Number of transcripts uniquely expressed in AD brain for unfiltered and machine learning (ML)-filtered alignment data

	Number of transcripts
Unfiltered alignment data	2729
ML-filtered alignment data	2663
Exclusive to unfiltered alignment data	136
Exclusive to ML-filtered alignment data	70

**Fig. 6:**
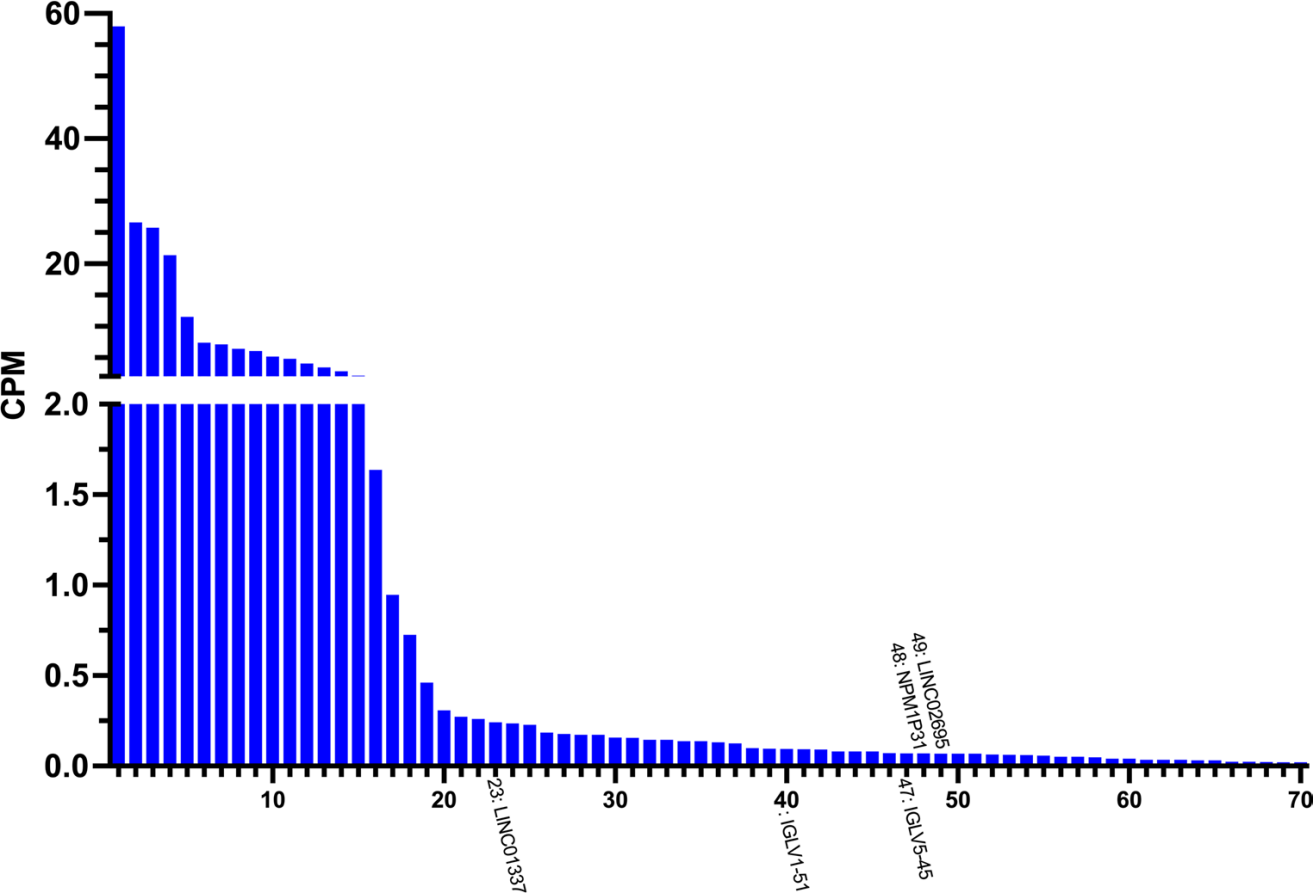
Distribution of expression levels of transcripts uniquely expressed in AD brain exclusive to machine learning-filtered alignment data. On the x-axis, the transcripts are ranked by their total expression across all samples in CPM (y-axis). Of these transcripts, 39 were highly expressed in one biological replicate, 20 in two replicates, seven in three, three in four, two in five, and one in six. Gene loci of transcripts involved in major pathological processes of AD and other neurodegenerative disorders are labelled correspondingly. The plot was created using Prism v10.2.0 for Mac, GraphPad Software, San Diego, California USA, www.graphpad.com

The 70 transcripts uniquely expressed in AD brain and exclusive to ML-filtered alignment data were subjected to further investigation. It was found that 15 transcripts exhibited a summed expression higher than 2 CPMs (Fig. 6, Table S4).

**Fig. 7:**
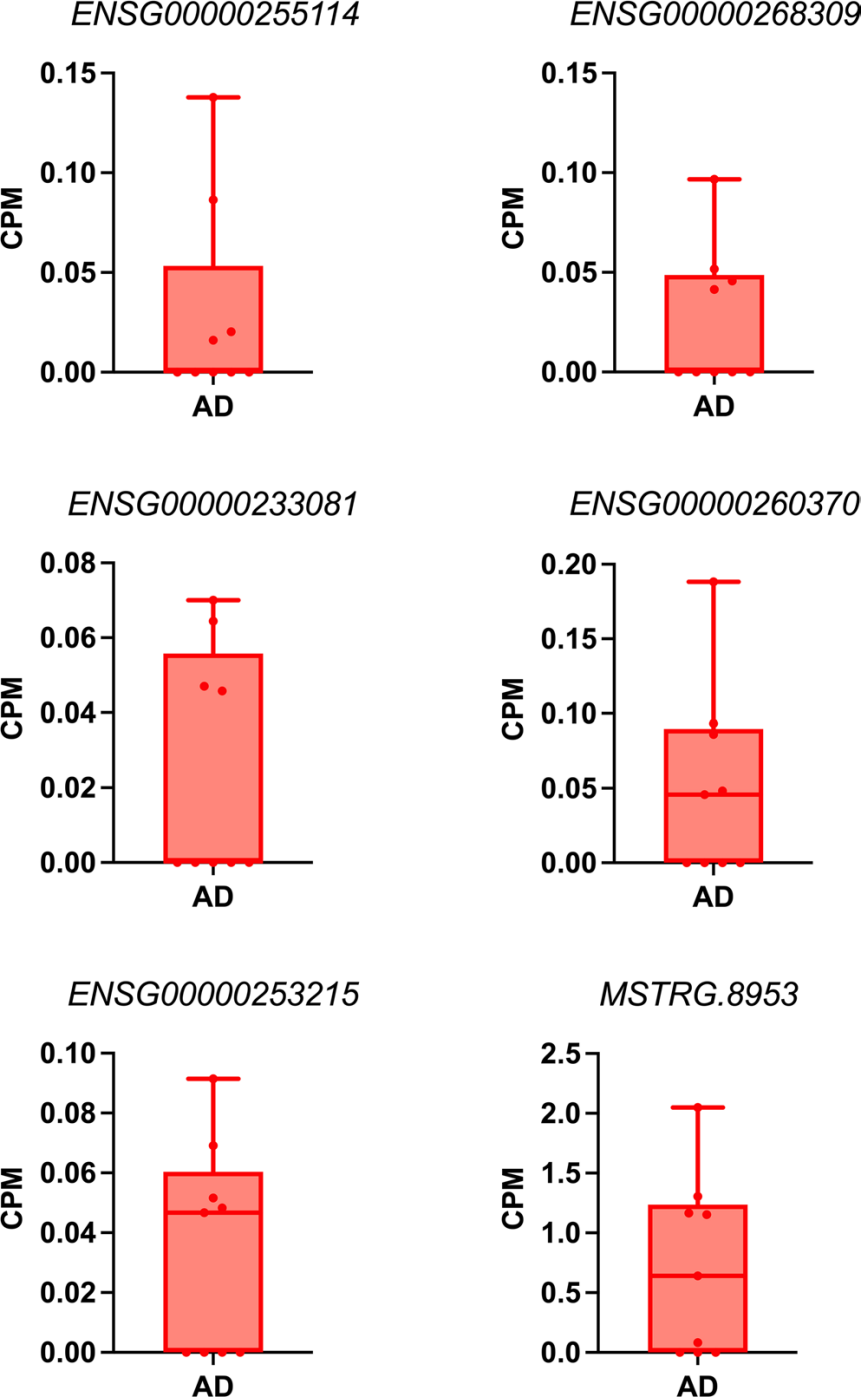
Transcripts uniquely expressed in AD brain exclusive to machine learning-filtered alignment data with expression in four or more biological replicates. *ENSG00000255114* (chr11:119044188–119045493), *ENSG00000268309* (chr19:16551773–16552328), and *ENSG00000233081* (chr9:91426238–91427144) were expressed in four replicates, *ENSG00000260370* (chr10:14138–20897) and *ENSG00000253215* (chr8:17882043–17882661) in five, and *MSTRG.8953* (chr12:68842197–68932884) in six. The “MSTRG” prefix indicates a novel transcript as determined by the transcript assembler, StringTie. The plot was created using Prism v10.2.0 for Mac, GraphPad Software, San Diego, California USA, www.graphpad.com

Out of the 70 transcripts, 39 were highly expressed in one biological replicate, 20 in two replicates, seven in three, three in four, two in five, and one in six (Fig. 7, Table S4). For the six transcripts exhibiting consistent expression in four or more biological replicates, five of them were determined to be long non-coding RNAs and one reported by the transcript assembler, StringTie, to be a novel transcript (Fig. 7).

During a manual literature search on the annotated gene loci of transcripts uniquely expressed in AD brain exclusive to ML-filtered alignment data (Fig. 6), it was discovered that the following genes were involved in major pathological processes of AD and other neurodegenerative disorders. According to the GeneCards human gene database (Stelzer et al. 2016), *IGLV1-51* and *IGLV5-45* were reported to be involved in immune response and antigen recognition; *LINC01337* was related to Huntington’s disease and cerebral cortex area; *LINC02695* implicated in AD and family history, as well as *NPM1P31* in Huntington’s disease and brain volume (Fig. 6).

The gene loci of transcripts uniquely expressed in AD brain shared between unfiltered and ML-filtered alignment data were examined. A total of 2,515 genes were found (Table S5). The consistency in detection suggests that these genes are robust candidates for downstream analyses.

### Differentially Expressed Alternative Splicing Events in Alzheimer’s Disease Brain

Using rMATS-turbo, DE alternative splicing events in control vs. AD brain were identified. Unlike DE analysis of transcripts, which captures changes in overall transcript abundance, rMATS-turbo detects DE alternative splicing events by measuring the abundance of splice junctions in reads that contain them. In unfiltered alignment data, 20,518 DE splicing events were detected, while in ML-filtered alignment data, 17,087 DE splicing events were identified. The gene loci associated with these DE alternative splicing events were then compared to the loci associated with the DE transcripts. This comparison may provide insights into the factors contributing to a transcript’s differential expression.

When compared to DE transcripts from unfiltered data, it was found that seven gene loci were commonly associated between the DE splicing events and DE transcripts: *HEATR5A*, *MBNL3*, *S100B*, *SORCS1*, *TBC1D10A*, *GEMIN8*, and *ENSG00000285218*. When compared to DE transcripts from ML-filtered data, six gene loci were shared: *HEATR5A*, *MBNL3*, *S100B*, *SORCS1*, *GEMIN8*, and *TBC1D10A*.

### Improved Alzheimer’s Disease Literature Relevance for Gene Loci Associated with Differentially Expressed Transcripts in Machine Learning-Filtered Data

An automated literature survey (see Materials and methods for developed algorithm details) was performed to assess the relevance of gene loci associated with DE transcripts in control vs. AD brain. Out of the 338 gene IDs identified from unfiltered alignment data, 48 (14.20%) have been documented in existing literature to be involved in AD or related neurobiological processes (Table 3, Table S6). In comparison, the ML-filtered approach produced 395 gene IDs, of which 63 (15.95%) were literature-relevant (Table 3, Table S7). There is a noticeable increase in the proportion of literature-supported gene IDs derived from DE transcripts in ML-filtered data compared to unfiltered data.

**Table 3:** Literature relevance of gene loci associated with DE transcripts in control vs. AD brain

	Unfiltered alignment data	Machine learning-filtered alignment data
Total number of gene IDs processed	338	395
Number of gene IDs identified with literature relevancy	48	63
The proportion of gene IDs with literature relevancy	14.20%	15.95%

The same literature survey method was applied to the 157 DE transcripts exclusive to ML-filtered alignment data (Table S1). The analysis revealed that 20 of these transcripts have been previously annotated in the literature as being correlated to AD and its related developmental processes (Table S8).

### More Enriched Gene Ontology Biological Processes Relevant to Alzheimer’s Disease for Gene Loci from Machine Learning-Filtered Data

GO enrichment analysis was conducted on gene loci associated with DE transcripts in control vs. AD brain. The analysis revealed significantly enriched GO biological processes (*p*-value < 0.05) in both unfiltered and ML-filtered alignment data (Table S9, Table S10).

Through examination of enriched GO biological processes, terms key to AD pathology were identified. In Table 4, these terms were categorised into: exclusive to unfiltered data/ML-filtered data, and common to both types of data, enabling a clear comparison of the GO results.

**Table 4:** Enriched AD-relevant gene ontology biological processes identified from gene loci of differentially expressed transcripts in AD brain for unfiltered and machine learning-filtered alignment data

	Exclusive to unfiltered data	Common to both types of data	Exclusive to machine learning-filtered data
1	GO:0001504 – neurotransmitter uptake	GO:0034349 – glial cell apoptotic process	GO:0005513 – detection of calcium ion
2	GO:0007218 – neuropeptide signaling pathway	GO:0051926 – negative regulation of calcium ion transport	GO:0007405 – neuroblast proliferation
3	GO:0048168 – regulation of neuronal synaptic plasticity	GO:0090279 – regulation of calcium ion import	GO:0042771 – intrinsic apoptotic signaling pathway in response to DNA damage by p53 class mediator
4	GO:2,000,425 – regulation of apoptotic cell clearance	GO:1,900,117 – regulation of execution phase of apoptosis	GO:0043523 – regulation of neuron apoptotic process
5			GO:0043524 – negative regulation of neuron apoptotic process
6			GO:0051402 – neuron apoptotic process
7			GO:0051924 – regulation of calcium ion transport
8			GO:0051928 – positive regulation of calcium ion transport
9			GO:0070997 – neuron death
10			GO:0072332 – intrinsic apoptotic signaling pathway by p53 class mediator
11			GO:0097150 – neuronal stem cell population maintenance
12			GO:1,901,214 – regulation of neuron death
13			GO:1,901,215 – negative regulation of neuron death
14			GO:1,902,253 – regulation of intrinsic apoptotic signaling pathway by p53 class mediator
15			GO:1,902,692 – regulation of neuroblast proliferation
16			GO:1,903,169 – regulation of calcium ion transmembrane transport
17			GO:1,903,170 – negative regulation of calcium ion transmembrane transport

GO terms exclusive to unfiltered alignment data primarily involved general neuronal signalling and synaptic processes (GO:0001504 – neurotransmitter uptake, GO:0007218 – neuropeptide signaling pathway) (Table 4). Terms shared between both types of alignment data were mostly associated with apoptotic regulation and calcium signalling (GO:0034349 – glial cell apoptotic process, GO:0051926 – negative regulation of calcium ion transport), suggesting that these pathways are robustly detected regardless of filtering (Table 4).

Notably, there was a broader and more specific set of apoptosis- and neurodevelopment-related terms found for ML-filtered alignment data, many of which were absent in the unfiltered dataset (Table 4). These terms included GO:0043523 – regulation of neuron apoptotic process, GO:0097150 – neuronal stem cell population maintenance, and several p53-mediated apoptotic pathways (e.g., GO:0042771, GO:0072332). The ML-filtered approach also captured nuanced regulation of calcium ion transmembrane transport (e.g., GO:1903169, GO:1903170) and neuroblast proliferation (GO:1902692).

**Fig. 8:**
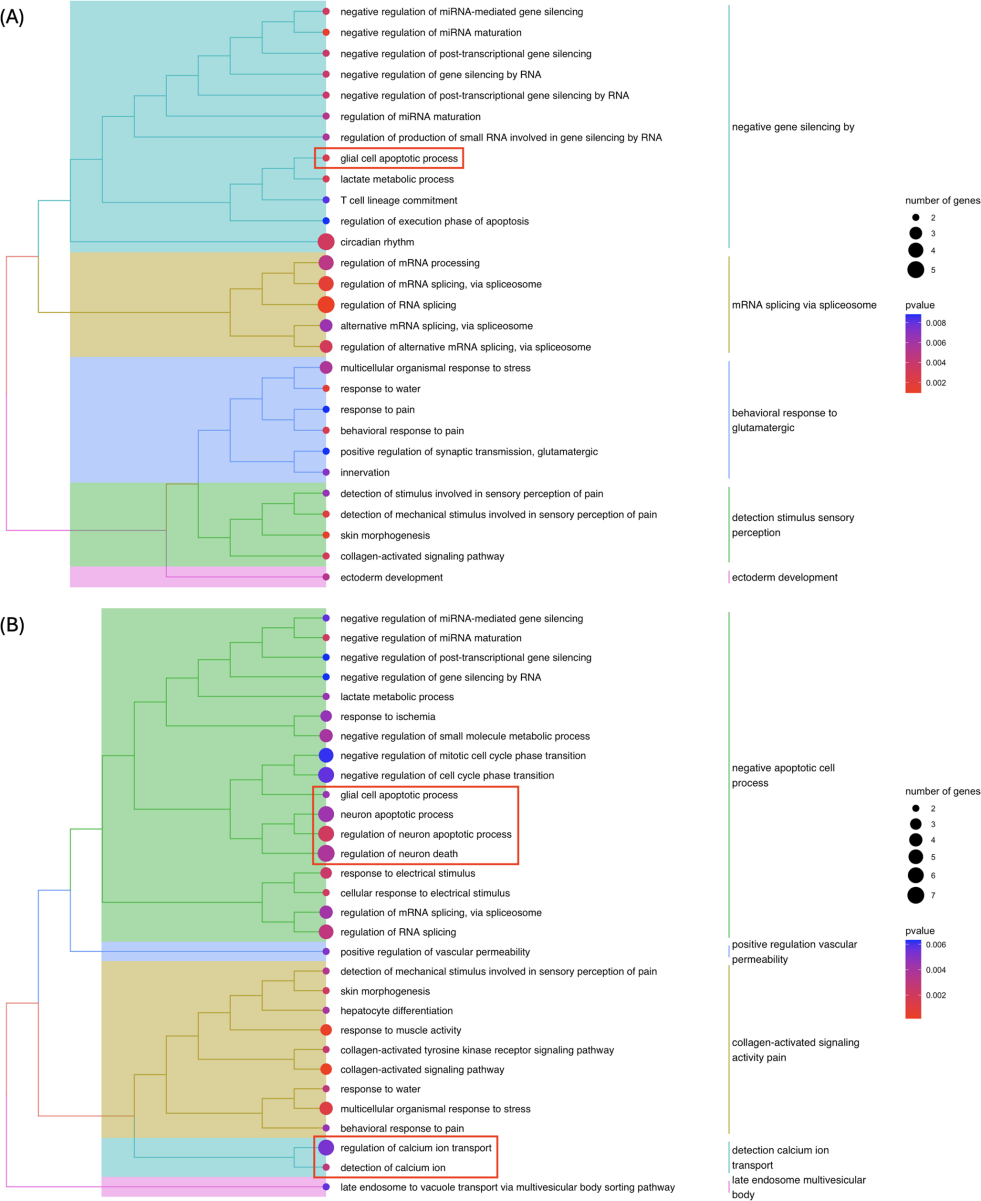
Enriched gene ontology (GO) biological processes for gene loci from DE transcripts in control vs. AD brain. (**A**) GO terms enriched from transcripts identified using unfiltered alignment data. (**B**) GO terms enriched from transcripts identified using machine learning–filtered alignment data. The figure was created using clusterProfiler’s *treeplot* function. Hierarchical clustering was performed using the average linkage method. Key GO biological processes relevant to AD are highlighted with red boxes. The figure displays the top 30 GO terms by statistical significance; for the full list, refer to Table S9 and Table S10

The increased representation of biological processes central to AD pathology in the ML-filtered alignment data is clearly illustrated in Fig. 8. This figure displays the top 30 GO terms by statistical significance. In the unfiltered alignment data (Fig. 8A), only one biological process key to AD pathology—glial cell apoptotic process—was shown. In contrast, the ML-filtered alignment data (Fig. 8B) exhibited six such biological processes, which form coherent clusters that are biologically relevant to one another.

Thirteen gene loci corresponding to DE transcripts exclusively found in ML–filtered alignment data (Table S1) contributed to the enrichment of key GO terms relevant to AD. These GO terms included keywords such as “neuro”, “apopto”, and “calcium”, reflecting critical biological processes implicated in AD pathology. The contributing genes were: *ANGPT1*, *CD44*, *CX3CR1*, *HHIP*, *LPAR4*, *MAPK8IP2*, *NAE1*, *PCDHGB4*, *PLP1*, *PROX1*, *REST*, *RYR2*, *SESTD1*.

### Novel Differentially Expressed Transcripts Exclusive to Machine Learning-Filtered Data Show Coding Potential and Cross-Species Conservation

To evaluate the potential biological relevance of novel DE MSTRG transcripts detected exclusively in ML-filtered data, coding potential prediction and cross-species conservation analyses were performed. A total of 62 novel MSTRG transcripts were examined. Using ORF-based coding potential analysis tool, CPC2, 29 transcripts (46.8%) were marked as coding and 33 transcripts (53.2%) as non-coding (Table S11). Those that were predicted to be coding transcripts typically consisted of long open reading frames with high coding probability scores, which are signs of protein-coding potential.

Evolutionary conservation was evaluated through BLASTn search against the mouse transcriptome, with the MSTRG transcript sequences as input. BLASTn result entries with high scores were identified for 39 of the 62 transcripts (62.9%), indicating high sequence conservation across species (Table S11). Importantly, conservation was strongly enriched among transcripts predicted to be coding. 25 out of 29 coding transcripts (86.2%) exhibiting long and high-identity matches to mouse transcripts with near-zero E-values (Table S11). This supports their classification as having potential biologically meanings rather than being artefacts. Such transcripts, include *MSTRG.1413.2*, *MSTRG.7529.1*, *MSTRG.8059.2*, and *MSTRG.38967.1*, contain high coding probability as well as convincing cross-species sequence conservation.

In contrast, a subset of transcripts (*n* = 13) displayed very low coding probability (< 0.1) and did not show detectable conservation in the mouse transcriptome. These transcripts were predicted to have only short ORFs and may be low-confidence transcript output or lineage-specific noncoding RNAs.

## Discussion

AD is a neurodegenerative disorder involving complex molecular mechanisms and progressive cognitive decline, necessitating large-scale multi-omic analyses (DeTure and Dickson 2019). Recent advances in sequencing technologies have led to the generation of vast amounts of data, often outpacing our ability to analyse them effectively, highlighting the need for powerful ML tools to extract meaningful insights (Berger and Yu 2023). While such tools offer significant potential, their performance is often assessed solely by the developers themselves, making independent evaluations both rare and critically important.

In this study, we investigated the effect of using an ML program to improve transcriptomic analytical pipelines, starting from the read alignment stage. The ML tool Splam, was employed to filter out low confidence splice junction reads, thereby refining alignment data derived from AD brain samples. We evaluated the impact of utilising Splam on downstream analyses, including DE analysis of transcripts and alternative splicing events, and identification of transcripts uniquely expressed in the disease condition. On gene loci associated with these transcripts of interest, the effect of Splam regarding their literature relevance to AD and GO enrichment analysis was also assessed. This was achieved through a series of comparative assessments that identified differences between the results of such analyses conducted on ML-filtered data and unfiltered data. In comparison to the non-ML approach, our results showed that using Splam led to a substantial increase in the number of DE transcripts and their corresponding gene loci with established relevance to AD. We also identified and examined transcripts uniquely expressed in AD, determined gene loci shared between DE transcripts and DE splicing events—enabling assessment of their possible connection—and observed more enriched GO biological processes related to AD using gene loci from DE transcripts.

We report an approximate 20% increase in the number of DE transcripts when using ML-filtered alignment data. Notably, the developers of Splam did not investigate the DE pattern of transcripts as a part of the program’s validation and no such analysis has been reported elsewhere to our knowledge. Thus, this increase in DE transcript detection represents a novel observation. One possible explanation is that the filtering of splice junction reads altered transcript abundance estimates in a way that amplified expression differences for certain transcripts, pushing them above the threshold for statistical significance. Given Splam’s mechanism—removal of low confidence or likely artifactual splice junction reads—it is plausible that noise reduction in transcript alignment led to more accurate quantification, effectively revealing biological differences that were previously obscured. This statement is supported by the 20 DE transcripts exclusive to ML-filtered data found to have disease relevance to AD as documented by existing literature (Table S8). The improvement likely stemmed from preventing the misassignment of reads to certain transcripts, i.e., reducing false-positive mappings, which led to more accurate estimates of transcript abundance (Srivastava et al. 2020). These findings suggest that ML-enhanced splice junction filtering can substantially influence downstream analyses, as shown by this difference in DE pattern between unfiltered and ML-filtered alignment data.

The DE analysis revealed that the proportion of up-regulated transcripts was substantially higher in comparison to down-regulated transcripts (Table 1). This observation corroborates with existing literature regarding AD brain. Marques-Coelho et al. (2021) reported 1,244 down- and 2,104 up-regulated genes using temporal lobe tissue of AD patients. There are studies that described patterns of more down-regulated transcripts, such as in Wu et al. (2023) and Gao et al. (2024)’s studies. An explanation for this could be that the former incorporated a fold-change cut-off, which this study did not apply, and the latter based its investigation on whole blood samples rather than brain. Other studies have also found approximately equal proportions of down- and up-regulated genes or transcripts (Hill and Gammie 2022; Yesudas et al., 2025).

Splam discarded approximately 10% of splice junction reads, which it deemed are of low confidence. Inspection of representative filtered reads using IGV revealed patterns suggestive of ambiguous or potentially non-canonical splicing events. Some reads appeared to span intronic regions or included both intronic and exonic segments, which may reflect partial intron retention, mis-annotation of exons, or limitations in the alignment algorithm when encountering complex regions of the transcriptome. These ambiguous splicing patterns were consistently observed across biological replicates.

Supporting these findings, junction-level quality assessment in a representative sample suggested that Splam-filtering mainly eliminated splice junction reads with features of low-confidence splicing. After filtering, the remaining set had far more junctions linked to standard GT–AG splice motifs, nearing the 90% mark reported in validated eukaryotic introns (Burset et al. 2000; Sheth et al. 2006). In parallel, a higher share of these junctions was backed by multiple sequencing reads, suggesting stronger evidence for real splicing events. The structural composition of inferred introns was also improved, with a drop in median intron length and the proportion of very long introns exceeding 100 kb, since such cases are rare naturally and often stem from false positives in short-read data (Mapleson et al. 2018). Moreover, post-filtering showed lower average junction counts per gene, aligning with reduced background noise due to alignment errors or inflated detection rates (Zhang et al. 2018).

While this cleaning of the alignment likely removes alignment artifacts and reduces noise in the data, it may also result in loss of biologically meaningful but low-abundance or unannotated splicing events, particularly in non-coding or poorly characterised regions of the transcriptome. Future studies should look to validate them using long-read sequencing technologies, which covers entire regions of splicing with less fragmentation. Nonetheless, Splam offers a cost-effective and efficient approach for enhancing short-read RNA-seq analyses by improving splice junction reliability in the context of complex transcript structures.

The pattern of DE transcripts and transcripts uniquely expressed in AD between unfiltered and ML-filtered data stayed largely the same. This suggested that the statistical algorithms for these analyses were fundamentally robust, and the ML approach was only making minor adjustments, without undermining the validity of previous investigations. In other words, removing potentially spurious or ambiguous splicing events did not substantially alter the core expression profile, reinforcing the biological relevance of the DE transcripts commonly identified in both unfiltered and ML-filtered data. This also helps in pinpointing biomarkers that are of high confidence and consistently expressed, as well as noise-like transcriptomic changes that do not contribute significantly to the DE profile of AD.

To investigate the biological relevance of transcripts uniquely expressed in AD and identified exclusively through ML-filtered data, a manual literature search was conducted. Of the 70 transcripts analysed, 61 originated from unannotated or poorly annotated genes. Among the remaining nine annotated genes, five had documented associations with AD pathology—*IGLV1-51* and *IGLV5-45*—were reported to be involved in immune response and antigen recognition; they are genes of the human immunoglobulin lambda light chain locus that contribute to the diversity of antibody antigen-binding sites (Hadzidimitriou et al. 2009; Gibson et al. 2023). The immune response, particularly neuroinflammation driven by activated microglia, is recognised as a key contributor to the development and progression of AD (Cai et al. 2022). Additionally, *IGLV1-51* has been documented to be DE in systemic extracellular vesicles from sporadic and familial AD patients (Villar-Vesga et al. 2020) and identified as a candidate biomarker using multiplex cerebrospinal fluid and serum proteomics (Liu et al. 2023).

*LINC01337* is a long intergenic non-protein coding RNA that was reportedly involved with gene variants associated with Huntington’s disease progression according to the GWAS Catalog database (Sollis et al. 2023). *LINC02695* is another of such non-coding RNA as recorded by GWAS Catalog. It has been documented to be down-regulated in AD by studies investigating gene signatures of AD and AD family histories (Sollis et al. 2023). In a Huntington’s disease study that analysed human glial progenitor cells transplanted into mice, *NPM1P31* was identified as a key DE gene (Vastrad and Vastrad 2025). These additional findings of gene loci that were not present in unfiltered data demonstrate that ML-filtered data reveals DE patterns previously obscured by alignment noise.

Gene loci commonly associated between DE splicing events and DE transcripts were identified to investigate the potential connection between the two types of DE patterns. Seven gene loci were determined from unfiltered alignment data: *HEATR5A*, *MBNL3*, *S100B*, *SORCS1*, *TBC1D10A*, *GEMIN8*, and *ENSG00000285218*. *HEATR5A* is a gene whose expression is altered in response to amyloid-β oligomer exposure, implicating it in early AD–related synaptic dysfunction (Sebollela et al. 2012). DE of *MBNL3* in the human brain through its involvement in the neuronal splicing alterations associated with AD progression has been previously observed (Marques-Coelho et al. 2021). The same observation for *MBNL3* was also found in microarray studies (Wong 2013). *S100B* belongs to a family of calcium-binding proteins that is upregulated in AD brain and is implicated in promoting neuroinflammation and astrocyte activation, contributing to disease pathogenesis (Cristóvão and Gomes 2019). Variants of *SORCS1* have been suggested to increase the risk of AD onset through pathways of amyloid precursor protein processing (Reitz et al. 2011). *TBC1D10A*, although not directly cited to be related to AD, was however, involved in GTPase regulator activity in response to the mitochondria-targeted antioxidant SkQ1 that supresses AD-like pathology progression (Stefanova et al. 2019). *GEMIN8* plays a role in the survival of motor neuron complex, linking it to myotrophic lateral sclerosis (Wei et al. 2024); it could have broader relevance to AD through shared mechanisms across neurodegenerative disorders. *ENSG0000028521* is poorly documented with no correlated AD literature and interestingly, it is the only gene locus excluded from the findings in the ML-filtered data. This suggests noise exclusion in the form of removing potentially biologically irrelevant DE gene loci. Our findings provide insights into the mechanisms of certain DE transcripts by identifying common gene loci shared with DE splicing events. The application of ML enhances this analysis by filtering out noise and emphasising biologically relevant patterns, thereby improving the interpretability and robustness of the identified connections.

Using an automated literature search algorithm, the lists of gene loci associated with DE transcripts were screened for AD relevance using a selection of keywords. We found that there was an increased ratio of genes with biological relevance to AD in ML-filtered data compared to unfiltered data. Although the improvement is modest, it is meaningful in that the filtering procedure occurred at an early stage and can thus have a large impact on all downstream analyses, as reflected in our findings.

Regarding the automated literature search algorithm, it allows for fast and consistent screening of gene relevance across large input lists, making it highly efficient and reproducible. However, the search strategy may miss relevant findings due to limited keyword scope and can include false positives from ambiguous or context-insensitive matches.

Using the same automated literature search algorithm, gene loci associated with DE transcripts exclusive to ML-filtered data were also investigated. A notable amount—20 out of 154—were found to have documented relevance to AD in existing literature. This demonstrates that ML-filtered data brings out additional biologically relevant transcripts of interest that may have been obscured by noise, reinforcing the fact that ML filtering treatment of the alignment influences downstream analyses in a positive way.

GO enrichment analysis was performed on the gene loci expressing DE transcripts. Notably, gene loci from the ML-filtered data yielded substantially more enriched biological processes that were related to AD pathology. This is especially evident from the number of AD-related terms enriched exclusively in ML-filtered data. These terms do not only encompass a greater range of neuron apoptosis regulation and calcium ion transport pathways, whose dysregulation are pivotal to the pathophysiology of AD (Ge et al. 2022; Goel et al. 2022), they also specify particular mechanisms of apoptosis; for example, GO:0042771, GO:0072332, and GO:1,902,253 are three biological processes enriched pointing specifically to the intrinsic apoptotic signalling pathway by p53 class mediator. This p53 class mediator apoptosis pathway has been documented to instigate neuronal apoptosis in response to DNA damage and oxidative stress (Culmsee and Mattson 2005), with oxidative stress being a prominent factor contributing to neurodegeneration in the form of atrophy (Breijyeh and Karaman 2020). Several studies have reported directly on the connection between p53 pathways and neurodegenerative diseases including AD and Parkinson’s disease (Abate et al. 2020; Wolfrum et al. 2022; Nelson and Xu 2023). It has been suggested that p53 contributes to neuronal dysfunction on the post-translational level through conformational misfolds (Abate et al. 2020).

The findings show that many of the gene loci and biological processes uncovered exclusively in the ML-filtered dataset align closely with the major pathological pathways recognised in AD. AD pathogenesis is driven by a number of interrelated mechanisms, the most prominent ones being amyloid-β (Aβ) dysregulation (Murphy and LeVine 2010), disruptions in tau-associated cytoskeletal processes (Jiang et al. 2025), chronic neuroinflammation (Lecca et al. 2022), early synaptic dysfunction (Pelucchi et al. 2022), and impairments in lipid metabolism (Yin 2023) and endosomal–lysosomal trafficking (Szabo et al. 2022).

Notably, many ML-specific GO terms match well-known biological processes quite precisely. For instance, functions linked to endosomal and lysosomal dynamics—such as *late endosome to lysosome transport* (Hu et al. 2015), *multivesicular body organization* (Von Bartheld and Altick 2011; Hu et al. 2015), and *receptor-mediated endocytosis* (Zadka et al. 2024). These biological processes directly implicate pathways essential for APP trafficking and Aβ turnover (Cam and Bu 2006; Jiang et al. 2014). Similarly, GO terms found to relate with cytoskeletal and centrosomal regulation, including *microtubule organizing center organization* (Li et al. 2007) and *centrosome cycle* (Granic and Potter 2013), are documented to cause disturbances in tau phosphorylation and microtubule stability observed in AD (Lovestone et al. 1996; Cyske et al. 2023). The enrichment of immune-related terms, particularly those involving microglial activation (Leng and Edison 2021; Valiukas et al. 2025) and interleukin-1β production (Shaftel et al. 2008), highlights how innate immune responses could worsen neuronal injury (Van Eldik et al. 2016; Butovsky et al. 2025). Synaptic processes such as *regulation of long-term synaptic potentiation* (Huh et al. 2016; Prieto et al. 2017) and *ionotropic glutamate receptor signalling* (Wang and Reddy 2017), alongside pathways involved in oxidative stress resistance (Zhao and Zhao 2013), including *regulation of oxidative stress-induced neuron and cell death* (Plascencia-Villa and Perry 2023), point to early neuronal dysfunction symptoms of AD. The lipid-related terms, including *cholesterol homeostasis* (Chang et al. 2017) and several processes in lipid transfer, absorption, and synthesis (Chew et al. 2020), contribute to APOE-mediated processes of Aβ aggregation and neuronal resilience (Chew et al. 2020; Hampel et al. 2021; Sprenger et al. 2025).

Moreover, numerous gene loci associated with DE transcripts found exclusively in the ML-filtered data were key contributors to the enrichment of GO terms highly relevant to AD (*ANGPT1*, *CD44*, *CX3CR1*, *HHIP*, *LPAR4*, *MAPK8IP2*, *NAE1*, *PCDHGB4*, *PLP1*, *PROX1*, *REST*, *RYR2*, *SESTD1*). A number of these loci are directly involved in established AD mechanisms. As an example, *CX3CR1* (Liu et al. 2025) and *CD44* (Matsumoto et al. 2012) are both closely connected to microglial activation and immune signalling in neuronal tissues, which are known to regulate Aβ clearance and inflammatory responses that can increase neuronal injury (Gao et al. 2023; Valiukas et al. 2025). *RYR2* (Yao et al. 2022) and *SESTD1* (Beech 2012), are implicated in calcium release and membrane signalling dynamics, which fits earlier findings showing disrupted calcium balance leads to synapse issues and increased neuron-related risk in AD (Supnet and Bezprozvanny 2010; Guan et al. 2021). Genes such as *MAPK8IP2*, which codes for the JIP2 protein (Roessler et al. 2018; Capilla-López et al. 2025), and *REST* (Khera et al. 2025) play roles in modulating neuronal stress responses, axonal transport, and synaptic homeostasis; their dysregulation have been reported to lead to early synaptic loss and impaired neuro-regenerative capability in AD (Cai and Tammineni 2017; Meftah and Gan 2023). Other loci, *PLP1* (Tatar et al. 2010) and *PROX1* (Gazestani et al. 2023), relate to oligodendrocyte function, myelination, and neuronal circuit maintenance, support existing literature on white matter damage and loss of myelin starting early in AD (Depp et al. 2023; Huang et al. 2024). Pathways consisting of *ANGPT1* (Peng et al. 2020) and *LPAR4* (Birgbauer 2024) further implicate vascular stability and lipid-mediated signalling, both of which have are important factors modulating Aβ deposition and neuroinflammatory susceptibility (Schreitmüller et al. 2012; Wang et al. 2025). Together, these ML-only gene loci highlight how the ML-filtered approach is better at picking up subtle but biologically meaningful gene expression changes. These changes point to key processes involved in AD, such as neuroinflammation, disrupted calcium signaling, synaptic problems, and myelin damage.

The additional genes and pathways uncovered through ML filtering may offer useful insight into AD progression and potential therapeutic targets. While AD is classically defined by Aβ and tau pathology, neuroinflammation, and synaptic loss, growing evidence suggests that a degree of biological plasticity is maintained throughout the course of the disease (Bhembre et al. 2023; Fan et al. 2025). One example of this plasticity is shown when restoring NF-α1/CPE expression in mouse models reduces amyloid quantity level, increases synaptogenesis and microglial activity, and improves cognition even at later stages (Fan et al. 2025). This indicated that certain signalling pathways could be targets of therapeutic intervention.

Several ML-exclusive genes are linked to pathways involved with this type of plasticity and neurotrophic signalling. For example, *MAPK8IP2*, *RYR2*, *LPAR4*, *PROX1*, and *REST* have been reported to play roles in MAPK activity, calcium release, lipid-related synaptic signalling, and neuronal transcriptional control (Roessler et al. 2018; Yao et al. 2022; Gazestani et al. 2023; Birgbauer 2024; Khera et al. 2025). Others, including *ANGPT1*, *CD44*, *CX3CR1*, *PLP1*, and *SESTD1*, are factors in processes of vascular stability, microglial communication, myelination, and membrane signalling (Tatar et al. 2010; Beech 2012; Matsumoto et al. 2012; Peng et al. 2020; Liu et al. 2025). Although not traditional neurotrophic factors, these genes are major factors in pathways that regulate neurotrophic responses, suggesting they may help support neuronal survival in a similar way to NF-α1/CPE. Enrichment for endosomal–lysosomal trafficking and receptor internalisation further marks pathways that affect APP processing, synaptic function, and neuroimmune regulation (Cam and Bu 2006; Hu et al. 2015; Birgbauer 2024), proposing potential directions in selecting new candidate molecules.

These results also connect to the current AD therapeutic landscape. Some commonly used treatments, such as acetylcholinesterase inhibitors, memantine, and agents with proposed neurotrophic effects, target neurotransmission and cognitive symptoms, with consistent yet modest effects (Fan et al. 2022). Certain ML-exclusive genes contribute to the pathways these drugs target. For instance, molecules involved in calcium and glutamatergic signalling (such as those coded by *RYR2* and *SESTD1*) may relate to processes affected by memantine (Goussakov et al. 2010; Yang et al. 2019), while genes linked to synaptic plasticity or microglial signalling (such as *LPAR4*, *CD44*, and *CX3CR1*) participate in pathways associated with neurotrophic-acting treatments (Matsumoto et al. 2012; Birgbauer 2024; Liu et al. 2025). Thus, several of the pathological pathways highlighted by the ML-filtered data, including endosomal trafficking, transcriptional stress responses, and myelination, while not currently direct targets of approved therapies, have potential in future therapeutic avenues.

Functional evaluation of the novel DE MSTRG transcripts detected only after ML filtering suggests that a substantial proportion are biologically meaningful rather than technical artefacts. Nearly half exhibit high coding potential based on ORF analysis, and almost all of these candidates are also conserved in the mouse transcriptome. Having both coding potential and evolutionary conservation makes them notable candidates for experimental validation, as protein-coding genes usually contain both long open reading frames and strong evidence of sequence constraint across mammals (Lin et al. 2011). These observations imply ML-filtering helps uncover undocumented transcripts resembling known gene features, thus broadening existing knowledge of genomic/transcriptomic references.

Meanwhile, some transcripts are predicted to have little coding ability and lack clear evolutionary conservation, which points to the underlying limitations of novel assemblies from RNA-seq data. Such transcripts could represent lineage-specific non-coding RNAs (Paralkar et al. 2014), or stem from technical issues linked to short-read sequencing, including shorter fragmentation and thus more likely spurious transcript reconstruction (Byrne et al. 2019). To be noted, predicting protein-coding capacity or relying on sequence conservation across organisms are not enough to confirm biological role; conservation patterns may simply indicate shared domains or paralogous relationships, while predicted open reading frames do not always lead to actual protein production (Li et al. 2020; Mishra and Wang 2021). Therefore, the results here offer a way to assign priority to novel transcripts instead of making definitive functional annotations. By integrating ML-based filtering with statistically independent computational evidence, this approach enables the identification of high-confidence novel transcript candidates for downstream experimental validation and functional investigation.

In conclusion, our findings demonstrate that using an ML program such as Splam to enhance the alignment data can improve the detection of biologically and disease-relevant transcriptional changes in the context AD. Compared to unfiltered data, the ML-filtered approach yielded a greater number of DE transcripts, a higher proportion of up-regulated signals, and more enriched GO terms directly related to AD pathology. Importantly, this method effectively filtered out potentially ambiguous or low confidence splicing events, improving the clarity and reliability of downstream analyses. Many of the gene loci identified exclusively in the ML-filtered dataset contributed to pathways central to neurodegeneration, including neuronal apoptosis, neurodevelopment, and calcium signalling.

Importantly, this study is one of few that independently tested an ML program in the context of a complex disease like AD. Most evaluations of ML tools, like Splam, come from the developers themselves, with reported external validations such as our study uncommon. To properly elucidate the effects of ML filtered data on downstream analyses in a variety of biological contexts, such studies incorporating these comparative analyses are needed, especially considering how the use of ML in bioinformatics is becoming more common. By applying this method to an independent AD transcriptomic dataset, we show the potential of ML tools to uncover clearer and more relevant biological signals, highlighting their practical value for disease-focused research.

## Supplementary Information

Below is the link to the electronic supplementary material.

Supplementary Material 1 (XLSX 15.3 MB)

## Data Availability

All data supporting the findings of this study are available within the paper and its Supplementary Information. Additional data are available from the corresponding author upon reasonable request.
